# Genetic and clinical risk factors for anti-tuberculosis drug-induced liver injury: insights from a prospective cohort study in central Ethiopia

**DOI:** 10.1007/s15010-025-02632-7

**Published:** 2025-09-04

**Authors:** Caroline Klindt, Andre Fuchs, Kristina Behnke, Carola Dröge, Kirsten Alexandra Eberhardt, Hans Christian Orth, Frieder Pfäfflin, Andreas Schönfeld, Tamara Nordmann, Million Getachew Mesfun, Verena Keitel, Tom Luedde, Tafese Beyene Tufa, Björn-Erik Ole Jensen, Torsten Feldt

**Affiliations:** 1 Department of Gastroenterology, Hepatology and Infectious Diseases, Medical Faculty, University Hospital Düsseldorf, Heinrich Heine University Düsseldorf, Düsseldorf, Germany; 2 Department of Medicine II, Medical Faculty, Mannheim of Heidelberg University, Mannheim, Germany; 3 Internal Medicine III – Gastroenterology and Infectious Diseases, University Hospital of Augsburg, Stenglinstr. 2, 86156 Augsburg, Germany; 4 Hirsch Institute for Tropical Medicine, Asella, Ethiopia; 5 Clinic for Gastroenterology, Hepatology and Infectious Diseases, Otto-von Guericke University, Leipziger Str. 44, 39120 Magdeburg, Germany; 6 Department of Infectious Diseases and Respiratory Medicine, Charité - Universitätsmedizin Berlin, corporate member of Freie Universität Berlin and Humboldt-Universität zu Berlin, Berlin, Germany; 7 Department of Infectious Diseases, West German Centre of Infectious Diseases, University Hospital Essen, University of Duisburg-Essen, Essen, Germany; 8 Department of Tropical Medicine, Department of Medicine, Bernhard Nocht Institute for Tropical Medicine, University Medical Center, Hamburg-Eppendorf, Hamburg, Germany; 9 Arsi University College of Health Science, Asella, Ethiopia

**Keywords:** Transient elastography, DILI, Single nucleotide polymorphism, Hepatobiliary transport, TB, Hepatotoxicity

## Abstract

**Purpose:**

Drug-induced liver injury (DILI) is a relevant adverse event of tuberculosis treatment (TBT) especially in sub-Saharan Africa, but data remains limited. Genetic hepatic transport proteins polymorphisms (HTPP) are potential contributors. This study aimed to assess frequency and timing of DILI, identify risk factors, and explore the association of HTPP with DILI risk in Ethiopian TBT-patients.

**Methods:**

In this prospective study, 424 confirmed tuberculosis patients in Ethiopian were recruited before initiation of TBT. Liver function tests were conducted during the first 8 weeks of treatment. Baseline evaluations included sociodemographic-, lifestyle- and clinical data including testing for viral co-infections, and HTPP as well as liver stiffness measurement by transient elastography (TE). Multivariable logistic regression, Cox proportional hazards models, and Fine and Gray competing risks analyses were employed for statistical analysis.

**Results:**

Cumulative DILI incidence was 16.0% with 4.2% classified as severe occurring most commonly within the first two weeks. Urban residence (OR 2.00, 95% CI 1.03–3.84; HR 1.80, 95% CI 1.00–3.22) was associated with increased DILI risk. In the competing risks model, urban residence (sHR 6.26, *p* = 0.010) and pathologic TE (sHR 5.23, *p* = 0.005) predicted severe DILI. The investigated HTPPs were not significantly associated with DILI.

**Conclusion:**

DILI is a common early complication of TBT in Ethiopian patients. Assessment of sociodemographic factors and TE before TBT may help identify high-risk individuals and offers a pragmatic approach for DILI management in resource-limited settings.

**Supplementary Information:**

The online version contains supplementary material available at 10.1007/s15010-025-02632-7.

## Introduction

Tuberculosis (TB) remains a major global health challenge, with 8 million people affected annually, predominantly in low- and middle-income countries (LMICs) [1–3]. In 2021, TB ranked as the 10th leading cause of death worldwide, with 87% of new cases occurring in just 30 high-burden countries. Ethiopia continues to be listed on the World Health Organization (WHO) High Burden Watch List, with an incidence rate of 126 cases per 100,000 population reported in 2022 [2, 3]. Contributing risk factors such as poor living conditions, malnutrition, human immunodeficiency virus (HIV) infection, and limited access to healthcare further increase TB prevalence and complicate treatment in these settings [4].

Effective TB treatment (TBT) requires a prolonged, uninterrupted multidrug regimen, most commonly including Isoniazid (INH), Rifampicin (RMP), Ethambutol (EMB), and Pyrazinamide (PZA). However, hepatotoxicity is a well-documented complication of this standard treatment for TB, with drug-induced liver injury (DILI) representing a major cause of treatment interruption, regimen modification, and - in severe cases - liver failure and death [5]. Hepatic adverse effects range from asymptomatic transaminase elevations to severe DILI and acute liver failure and most commonly have a hepatocellular injury pattern [6]. Several host-related risk factors have been described, including advanced age, malnutrition, female sex, concomitant infections such as HIV and chronic hepatitis B (HBV) as well as pre-existing liver disease, alcohol use, or concomitant use of other hepatotoxic drugs [7, 8]. Regular monitoring of liver function tests is therefore recommended [9]. In high-burden LMICs such as India, TBT is the leading cause of DILI, suggesting that similar patterns of TBT-associated DILI may be present in other LMICs with high TB burden [10]. Globally, the estimated incidence of DILI among patients receiving TBT ranges from 2.5 to 28%, though data on DILI incidence in the Ethiopian population are limited due to low recruitment numbers and inconsistent results [8]. Previous studies on DILI and associated risk factors in Ethiopia have described a DILI incidence between 8 and 30% [11–13]. A South African study reported an in-hospital and 3-month mortality rate of 27% in patients with TBT-associated DILI, underscoring the clinical relevance of early diagnosis and prevention [14]. The underlying mechanisms of DILI are incompletely understood but are thought to involve toxic metabolite formation, oxidative stress, and immune-mediated responses [8]. Due to the lacking in-depth comprehension of DILI, no effective preventive therapies currently exist [15, 16]. Particularly in the Ethiopian population with an estimated prevalence of viral hepatitis in the general population ranging from 2.1 to 25% and malnutrition being a common occurrence with approximately 21% of children being underweight, DILI under TBT is a relevant concern [7, 8, 17, 18]. Previously, comorbidities such as HIV co-infection, malnutrition, concomitant hepatotoxic medication, alcohol consumption and old age have been reported as relevant risk factors for DILI in Ethiopia [11–13, 19, 20]. However, these factors alone do not fully explain the variation in individual susceptibility. Moreover, the limited availability of reliable epidemiological data from high-burden countries hampers the development of effective monitoring and prevention strategies.

In recent years, genetic predisposition has emerged as an additional risk factor for DILI. Previous studies have identified certain Human Leukocyte Antigen such as HLA-DPB1*05:01 and HLA-B*57 variant alleles as potential contributors to DILI susceptibility [21, 22]. In different studies conducted in European and Asian populations, single nucleotide polymorphism (SNPs), gene variants with a minor allele frequency of > 1%, in genes encoding hepatic transporter proteins have been shown to play an important role in DILI susceptibility. Variants in genes encoding hepatobiliary transport proteins, such as bile salt export pump (BSEP, *ABCB11*), multidrug resistance protein (MDR) 3 (*ABCB4*), and MDR1 (*ABCB1*), play a crucial role in hepatic detoxification and bile formation [23]. While transporter gene variants may be clinically silent in healthy individuals, their impact becomes pronounced during prolonged exposure to hepatotoxic agents such as INH and RMP [15, 24, 25]. Notably, variants in *ABCB4* can result in a complete loss of protein expression or can be minor, leading to a broad clinical spectrum of *ABCB4*-associated diseases - from severe progressive familial intrahepatic cholestasis type 3 with early development of liver cirrhosis and the need for liver transplantation, to less severe diseases such as low phospholipid-associated cholelithiasis, intrahepatic cholestasis of pregnancy, or DILI. Some individuals may even remain clinically inapparent [26]. Interestingly, *ABCB4* mutations have been found to be more prevalent in the African-American population and are associated with both intrahepatic cholestasis of pregnancy and DILI, suggesting a relevant impact on the Ethiopian population as well [24]. Additionally, a previous study in Ethiopia has identified the *ABCB1* variant rs1045642 as a risk factor for DILI under TBT [27]. The injury pattern most commonly observed in TBT-associated DILI is hepatocellular in about 71% of cases, while the remaining cases have an almost equal distribution of cholestatic or mixed injury patterns. Currently, it is unclear if there are predetermining factors influencing the pattern of injury and different variants of biliary transport proteins might be a contributing factor. For instance, patients with deficiency in BSEP usually have a hepatocellular injury type, as reduced BSEP-transporter capacity leads to an accumulation of toxic metabolites inside of the hepatocyte and could predispose to a hepatocellular injury type under TBT, whereas reduced activity of MDR1 and MDR3 leads typically to a cholestatic liver injury pattern [6]. However, data on the prevalence and clinical significance of these variants in sub-Saharan Africa remain limited.

To our knowledge, there is limited information on the incidence of TBT-associated DILI in Ethiopia and few studies have evaluated the role of hepatic transporter gene variants in this context. This prospective cohort study aimed to systematically investigate the incidence of DILI in Ethiopian patients receiving standard TBT, to identify relevant clinical, socioeconomic, and genetic risk factors for DILI – including variants in key hepatobiliary transporter genes – and to provide evidence for improved monitoring and potential prophylactic strategies, such as ursodeoxycholic acid (UDCA) in genetically predisposed individuals [16]. The findings of this study are expected to enhance the understanding of DILI pathophysiology in high-burden settings and support the development of personalized, risk-adapted approaches to TB treatment.

## Methods

### Ethical considerations

Ethical approval has been obtained by the respective institutional ethical review board of the Adama Science and Technology University, Ethiopia (reference number A/h/S/S/7/120/11.592/06), the National Ethical Review Board of the Ethiopian Ministry of Science and Technology in Addis Ababa, Ethiopia (reference number 310/036/2015), and the Ethics Committee of the Faculty of Medicine, Heinrich-Heine-University, Düsseldorf, Germany (study number: 5028). After explanation of study procedures in mother tongue, written informed consent was obtained from all participating patients or legal guardians (where appropriate) before inclusion. The study was conducted according to the principles of the Declaration of Helsinki.

### General information

**Fig. 1:**
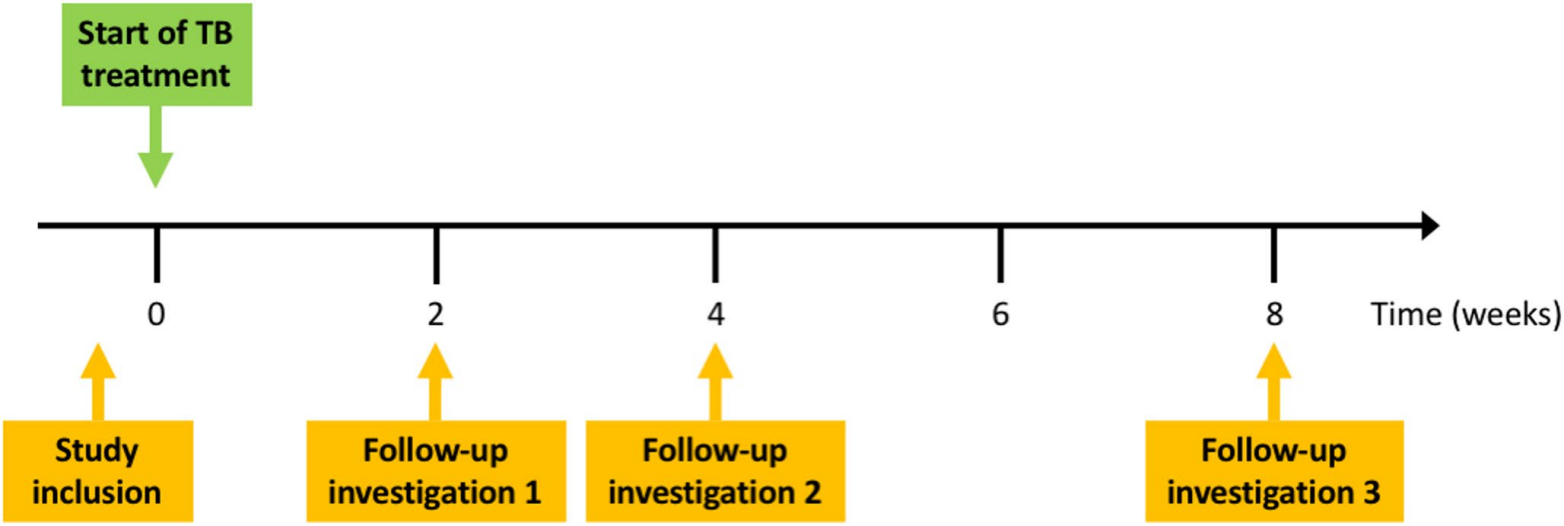
Timeline of study investigations. TB: tuberculosis;

This prospective cohort study on TBT-associated DILI was conducted over a period of 21 months in 2014/15 among patients newly diagnosed with TB at the Asella Referral and Teaching Hospital in Asella, Ethiopia, as well as at five affiliated health centers in the surrounding region. Patients were eligible for inclusion if they had a new diagnosis of TB and a planned start of standard quadruple TBT according to the Ethiopian national guidelines (Suppl. Table 1), within two weeks after study enrollment (Fig. 1) [5]. Patients were excluded if they had already initiated TBT prior to inclusion and baseline screening, had received TBT within the past six months, were pregnant, or were unable or unwilling to give informed consent.

### Recruitment and baseline investigations

All participants meeting the inclusion criteria underwent a baseline investigation at the time of study enrollment (Fig. 1), following a standardized protocol. Clinical and sociodemographic data were acquired through structured interviews, with a brief clinical examination for signs of chronic liver disease (CLD) such as jaundice, spider angiomas, palmar erythema, and enlargement of liver and/or spleen.

Liver stiffness was determined by transient elastography (TE) using a Fibroscan^®^ 402 (Echosens, Paris, France) following the manufacturer’s instructions. Measurements with at least 10 successful acquisitions, a success rate of ≥ 60% and an interquartile range (IQR) of < 30% of the median value were considered valid. Values of 7.65 kPa or higher were considered indicative of liver fibrosis (Metavir score ≥ F2), while values ≥ 13 kPa were classified as liver cirrhosis (Metavir score F4) [28]. Values between 6.0 kPa and 7.64 kPa were defined as intermediate and therefore not included in this comparison.

A venous blood sample was drawn from all participants for laboratory analyses, including liver function tests (LFTs), full blood count, and genetic polymorphism analysis of hepatobiliary transport proteins. The LFTs comprised aspartate transaminase (AST), alanine transaminase (ALT), and total bilirubin. If baseline evaluation revealed signs of chronic liver diseases or elevated liver enzymes, rapid tests for HBV surface antigen and HCV antibodies (different manufacturers according to local availability) were performed.

### Follow-up investigations and definition of drug-induced liver injury

Clinical and laboratory follow-ups were conducted at 2, 4, and 8 weeks after initiation of TBT (Fig. 1). Only new but no cumulative DILI cases are being displayed at each individual time point. Each follow-up included venous blood sampling for LFTs and a standardized interview to assess adherence to TBT and the presence of side effects. Endpoints were incidence of DILI and severe DILI according to laboratory test results, interruption of TBT, re-initiation of TBT, severe clinical complications like liver failure or death. DILI and severe DILI were identified based on the clinical chemistry criteria established by the Council for International Organizations of Medical Sciences working group on DILI (Table 1): DILI was present if ALT or AST were equal to or above twice the upper limit of normal (ULN). Severe DILI was defined as ALT or AST at least five times the ULN, or at least three times the ULN together with total bilirubin at least twice the ULN [29]. For interpretation, the following ULNs were applied: ALT 32 U/L, AST 31 U/L, and total bilirubin 1.0 mg/dL. In case of abnormal liver biochemistry prior to starting treatment with the implicated drug, ULNs were replaced by the mean baseline values obtained prior to the exposure to the suspect drug.

**Table 1:** Council for international organizations of medical sciences definitions of drug-induced liver injury applied [21]; DILI: drug-induced liver injury; ALT: Alanin aminotransferase; AST: asparate aminotransferase; FU: follow-up; ULN: upper limit of the normal range; B: baseline

Liver function tests		
Baseline	DILI at Follow-up 1, 2, or 3	
	Any form	Severe DILI
ALT or AST within normal range	ALT_FU_ or AST_FU_ >2x ULN	ALT_FU_ or AST_FU_ >5x ULN *or* ALT_FU_ >3x ULN *and* total Bilirubin_FU_ ≥2x ULN
ALT or AST > ULN	ALT_FU_ >2x ALT_B_ or AST_FU_ >2x AST_B_	ALT_FU_ >5x ALT_B_ or AST_FU_ >5x AST_B_

### Genetic polymorphisms of the hepatobiliary transporter proteins

Using QIAamp^®^ DNA blood mini kits (QIAGEN GmbH, Venlo, Netherlands), DNA was extracted from whole blood samples following manufacturer’s instructions. Genes of the hepatobiliary transport proteins BSEP, MDR1, and MDR3 were investigated for SNPs known to have an impact on transporter capacity, using TaqMan^®^ SNP Genotyping assays (Applied Biosystems, Waltham, MA, USA) provided in Suppl. Table 2 as described previously [30].

### Data handling and statistical analysis

Continuous variables were summarized as median and IQR and compared using the Kruskal-Wallis test, while categorical variables were analyzed with Fisher’s exact test. Pairwise post hoc tests with false discovery rate (FDR) correction were applied for variables showing statistical significance. A tiered approach was used to analyze DILI risk factors: logistic regression was performed first, followed by Cox proportional hazards models to incorporate temporal event dynamics. Ordinal regression models explored DILI severity, and Fine-Gray subdistribution hazard models accounted for competing risks in time-to-event analyses. Significance was set at *p* < 0.05. Analyses were conducted in R (version 4.4.3; R Foundation for Statistical Computing, Vienna, Austria).

## Results

Over an 18-month period, 438 patients diagnosed with TB were recruited prior to the initiation of TBT. Patients with a fatal outcome (*n* = 7) or who were lost to follow-up (*n* = 7) were excluded from the analysis, resulting in a final cohort of 424 participants. Sensitivity analyses assigning them to either the non-DILI group or the severe DILI group did not alter the key outcomes in risk analyses.

### Incidence of DILI

**Fig. 2:**
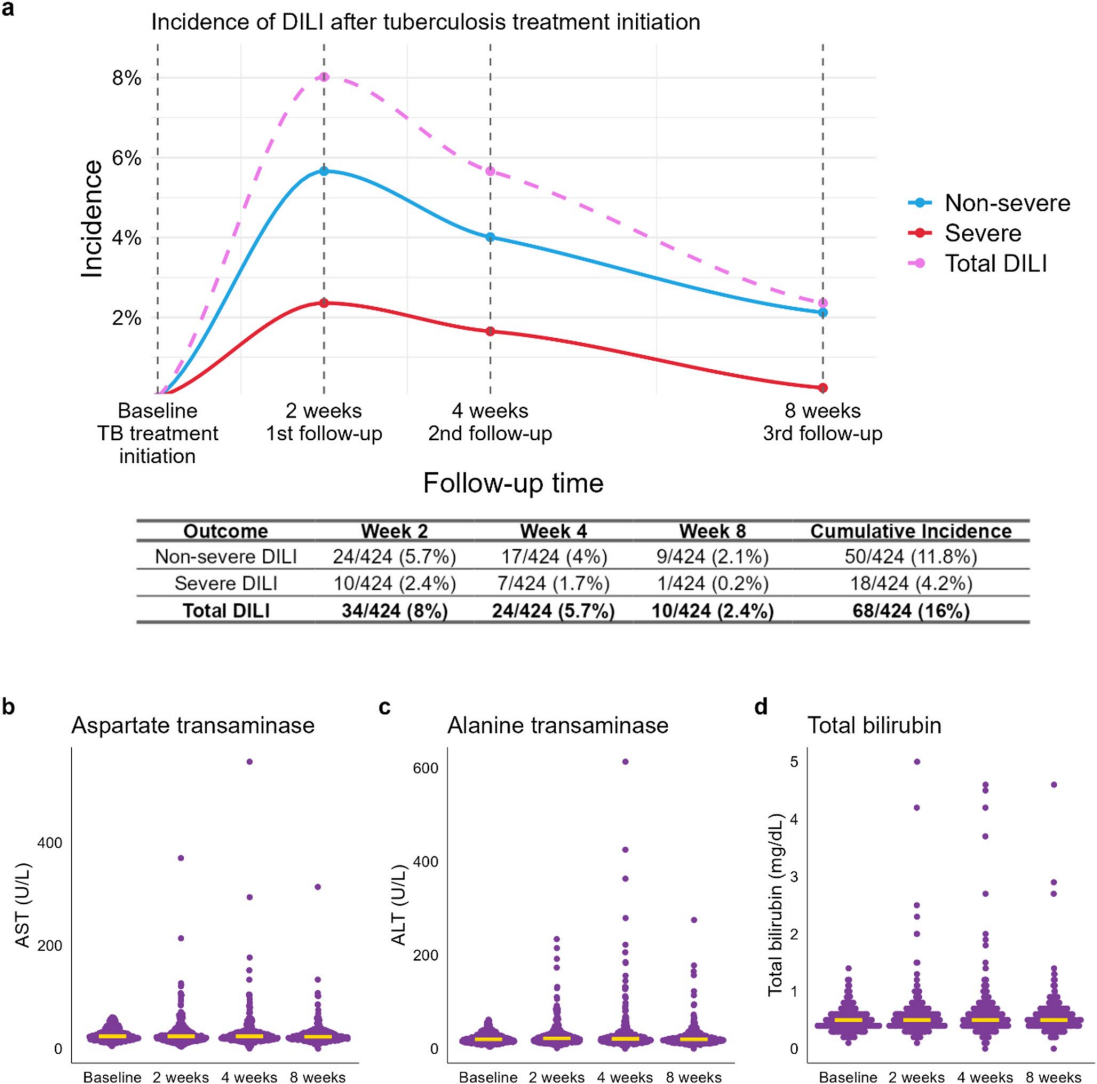
(**a**) Incidence of non-severe (*n* = 50), severe (*n* = 18), and total DILI cases (*n* = 68) during the 8-week observation period following initiation of tuberculosis treatment, assessed at three defined time points (weeks 2, 4, and 8). Only new DILI cases are displayed. DILI: drug-induced liver injury; TB: tuberculosis; (**b**) LFTs in patients with normal baseline levels during the observation period. Medians are displayed as horizontal lines

The incidence of DILI in the study population was 8.0% at week 2, decreasing slightly to 5.7% at week 4, and further declining to 2.4% by the 8-week follow-up visit, resulting in a cumulative incidence of 16.0% (Fig. 2a). Severe DILI occurred in 2.4% of patients at week 2, 1.7% at week 4 and 0.2% by week 8, with a cumulative incidence of 4.2% (Fig. 2a). Although median values of liver enzymes did not change after treatment initiation, Figs. 2b-d display elevations in affected patients during the follow-up period (Suppl. Table 3).

### Baseline characteristics of the study population

**Fig. 3:**
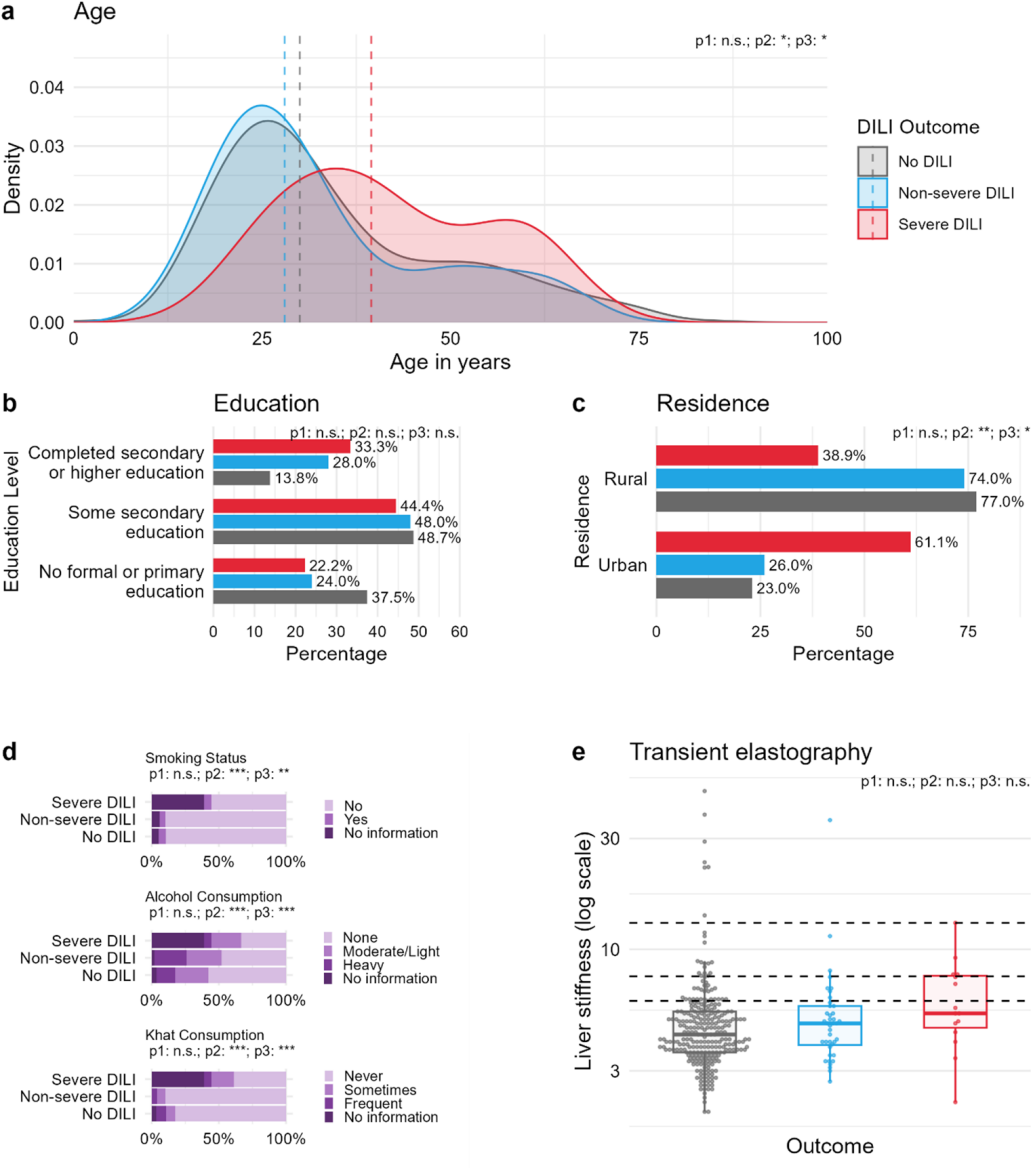
Demographic and clinical baseline characteristics according to occurrence of DILI. p1, p2, and p3 indicate pairwise comparisons: p1: no DILI (grey color) vs. non-severe DILI (blue color); p2: no DILI vs. severe DILI (red color); and p3: non-severe DILI vs. severe DILI. Significance is marked as * for 0.05, ** for *p* < 0.01, *** for *p* < 0.001, and n.s. for not significant (*p* ≥ 0.05). Annotations display which group differences are significant after multiple testing correction (method: False discovery rate). a: Distribution of age in years. Vertical dashed lines indicate median values of outcome groups. b: Ethnicity of participants according to DILI occurrence and severity. c: Educational status of participants. Completed secondary or higher education corresponds to a minimum education time of 11 years, while some secondary education is defined as five to 10 years of education. Four or less educational years and illiteracy are presented as no formal or primary education. d: Residence area according to outcome groups. e: Self-reported smoking, alcohol, and Khat consumption. Moderate/Light alcohol consumption corresponds to one to five drinks/week while heavy stands for more than five drinks/day. Sometimes as answer to Khat use is defined as in average once a week, while frequent stands for twice or more often/week. f: Liver stiffness measurements displayed on a log-scale for each outcome group. Horizontal dashed lines indicate cutoff values for the definition of non-pathological measurements (< 6 kPa), intermediate values (6-7.64 kPa), liver fibrosis (7.65–12.99 kPa) and liver cirrhosis (≥ 13 kPa). DILI: drug-induced liver injury;

The study population had an almost equal gender distribution with 43.2% females and 56.8% males (Table 2). Significant differences in baseline characteristics were observed across DILI severity groups. Patients with severe DILI were older (median 40 years; IQR 30–57) compared to those with non-severe DILI (28 years; IQR 22–40) and no DILI (30 years; IQR 23–45; *p* = 0.031, Fig. 3a-c). Residence in urban areas was more frequent among patients with severe DILI (61.1%) than in those with non-severe (26.0%) or no DILI (23.0%; *p* = 0.003). Differences in the formal education level of participants were also statistically significant (*p* = 0.019), although pairwise comparisons did not reveal significant differences between specific groups after FDR correction (Table 2).

**Table 2:** Demographic parameters according to occurrence of drug-induced liver injury. DILI: drug-induced liver injury; FDR: false discovery rate;

Variable	all	No DILI	Non-severe DILI	Severe DILI	*p*-value	FDR corrected post hoc *p*- values
Age in years	30 (23/45)	30 (23/45)	28 (22/40)	40 (30/57)	0.031	0 vs. 1: 0.443; 0 vs. 2: 0.021; 1 vs. 2: 0.021
Sex					0.935	
Female	183 (43.16%)	155 (43.54%)	21 (42%)	7 (38.89%)		
Male	241 (56.84%)	201 (56.46%)	29 (58%)	11 (61.11%)		
Residence					0.003	0 vs. 1: 0.721; 0 vs. 2: 0.003; 1 vs. 2: 0.016
Urban area	106 (25%)	82 (23.03%)	13 (26%)	11 (61.11%)		
Rural area	318 (75%)	274 (76.97%)	37 (74%)	7 (38.89%)		
Education					0.019	0 vs. 1: 0.070; 0 vs. 2: 0.102; 1 vs. 2: 0.938
No formal or primary education	149 (35.22%)	133 (37.46%)	12 (24%)	4 (22.22%)		
Some secondary education	205 (48.46%)	173 (48.73%)	24 (48%)	8 (44.44%)		
Completed secondary or higher education	69 (16.31%)	49 (13.8%)	14 (28%)	6 (33.33%)		

Significant differences in self-reported lifestyle factors were observed across DILI outcome groups (Fig. 3d; Table 2). Smoking status differed significantly (*p* = 0.001), with the severe DILI group having a notably higher proportion of participants who did not provide information (38.9%) and a lower proportion of non-smokers (55.6%) compared to the no DILI (5.1% and 89.6%) and non-severe DILI groups (6.0% and 90.0%, respectively). Alcohol consumption also showed significant variation (*p* = 0.001), with the severe DILI group reporting a lower percentage of abstainers (33.3%) and a higher proportion of participants not answering this question (38.9%) relative to the other groups (57.6% and 3.7% in the no DILI group and 48.0% and 2.0% in the non-severe DILI group). Similarly, Khat use was significantly associated with DILI outcomes (*p* = 0.001); the severe DILI group had fewer participants reporting “Never” (38.9%) and a higher percentage of missing responses (38.9%) compared to other groups (82.6% and 3.4% in the no DILI group and 90.0% and 0% in the non-severe DILI group, respectively). Post hoc pairwise comparisons revealed significant differences mainly between the group without the occurrence of DILI and the severe DILI group.

No differences were observed across DILI groups for infections with HBV, HCV, HIV, or previous TBT (Suppl. Table 5). Significantly higher proportions of participants in the severe DILI group did not provide information on known chronic or liver diseases or intake of drugs (Suppl. Table 5).

All patients underwent TE for liver stiffness measurement before initiation of TBT (Fig. 3e). Valid TE results were available for 298 patients. A total of 6.4% and 3.0% of all patients had a TE-value indicating fibrosis and cirrhosis, respectively. The proportion of participants with pathologic liver stiffness was higher in the severe DILI group (30.8%) compared to the no DILI (8.8%) and non-severe DILI (8.6%) groups with median values of 4.3, 4.8, and 5.3 kPa in the no DILI, non-severe DILI, and severe DILI groups, respectively (*p* = 0.035). However, pairwise comparisons between groups did not reach statistical significance (*p* = 0.258, 0.051, and 0.141). No significant differences were observed in the clinical pattern of injury between the non-severe and severe DILI group (31.9% vs. 55.6% hepatocellular pattern, 48.9% vs. 33.3% mixed pattern, 19.2% vs. 11.1% cholestatic pattern, respectively) [31].

### Polymorphisms in hepatic canalicular transporters

In the total study population, the majority of participants carried the reference allele (Table 3, Suppl. Table 2) for the assessed gene variants. Specifically, for BSEP rs3815676, 92.9% were positive for AA 6.6% AG, and 0.5% for the variant allele GG. For BSEP *rs7577650*, 14.6% carried the CC, 48.4% CT, and 37.0% the minor TT allele. For the BSEP V444A variant (*rs2287622*) 22.4% were expressing the reference allele TT, 49.5% TC, and 28.1% CC. The MDR1 SNP *rs1045642* was present in 60.8% as the major TT allele, in 34.0% TC, and in 5.2% as the minor CC allele. For the MDR3 polymorphisms, frequencies of the reference sequence ranged from 47.6 to 50.5%, heterozygous from 39.3 to 42.4%, and polymorphisms from 9.4 to 11.3%, depending on the locus. No significant differences in genotype distribution were observed across the DILI outcome groups (all *p* > 0.25, Table 3).

**Table 3:** Polymorphisms in hepatic canalicular transporters according to outcome groups (*DILI = drug-induced liver injury; FDR: false discovery rate; BSEP: bile salt export pump; T: thymine; A: adenine; C: cytosine; G: guanine; MDR: multidrug resistance protein*)

SNP Allele	All	No DILI	Non-severe DILI	Severe DILI	*p*	FDR corrected post hoc *p* values
BSEP rs3815676					0.697	0 vs. 1: 0.992; 0 vs. 2: 0.992; 1 vs. 2: 1.000
AA	394 (92.92%)	328 (92.13%)	48 (96%)	18 (100%)		
AG	28 (6.6%)	26 (7.3%)	2 (4%)	0 (0%)		
GG	2 (0.47%)	2 (0.56%)	0 (0%)	0 (0%)		
BSEP rs7577650					0.732	0 vs. 1: 0.752; 0 vs. 2: 0.752; 1 vs. 2: 0.752
CC	62 (14.62%)	51 (14.33%)	8 (16%)	3 (16.67%)		
CT	205 (48.35%)	171 (48.03%)	27 (54%)	7 (38.89%)		
TT	157 (37.03%)	134 (37.64%)	15 (30%)	8 (44.44%)		
BSEP V444A rs2287622					0.431	0 vs. 1: 0.801; 0 vs. 2: 0.385; 1 vs. 2: 0.385
TT	95 (22.41%)	83 (23.31%)	10 (20%)	2 (11.11%)		
TC	210 (49.53%)	173 (48.6%)	24 (48%)	13 (72.22%)		
CC	119 (28.07%)	100 (28.09%)	16 (32%)	3 (16.67%)		
MDR1 rs1045642					0.730	0 vs. 1: 0.847; 0 vs. 2: 0.847; 1 vs. 2: 0.847
TT	257 (60.76%)	218 (61.41%)	27 (54%)	12 (66.67%)		
TC	144 (34.04%)	117 (32.96%)	21 (42%)	6 (33.33%)		
CC	22 (5.2%)	20 (5.63%)	2 (4%)	0 (0%)		
MDR3 rs2109505					0.903	0 vs. 1: 0.865; 0 vs. 2: 0.865; 1 vs. 2: 0.865
AA	202 (47.64%)	169 (47.47%)	26 (52%)	7 (38.89%)		
AT	174 (41.04%)	146 (41.01%)	19 (38%)	9 (50%)		
TT	48 (11.32%)	41 (11.52%)	5 (10%)	2 (11.11%)		
MDR3 rs2302386					0.670	0 vs. 1: 0.716; 0 vs. 2: 0.716; 1 vs. 2: 0.716
TT	214 (50.47%)	180 (50.56%)	27 (54%)	7 (38.89%)		
TC	170 (40.09%)	140 (39.33%)	20 (40%)	10 (55.56%)		
CC	40 (9.43%)	36 (10.11%)	3 (6%)	1 (5.56%)		
MDR3 rs4148826					0.269	0 vs. 1: 0.326; 0 vs. 2: 0.326; 1 vs. 2: 0.326
AA	194 (45.75%)	162 (45.51%)	27 (54%)	5 (27.78%)		
AG	180 (42.45%)	149 (41.85%)	20 (40%)	11 (61.11%)		
GG	50 (11.79%)	45 (12.64%)	3 (6%)	2 (11.11%)		

### Factors associated with drug-induced liver injury

**Fig. 4:**
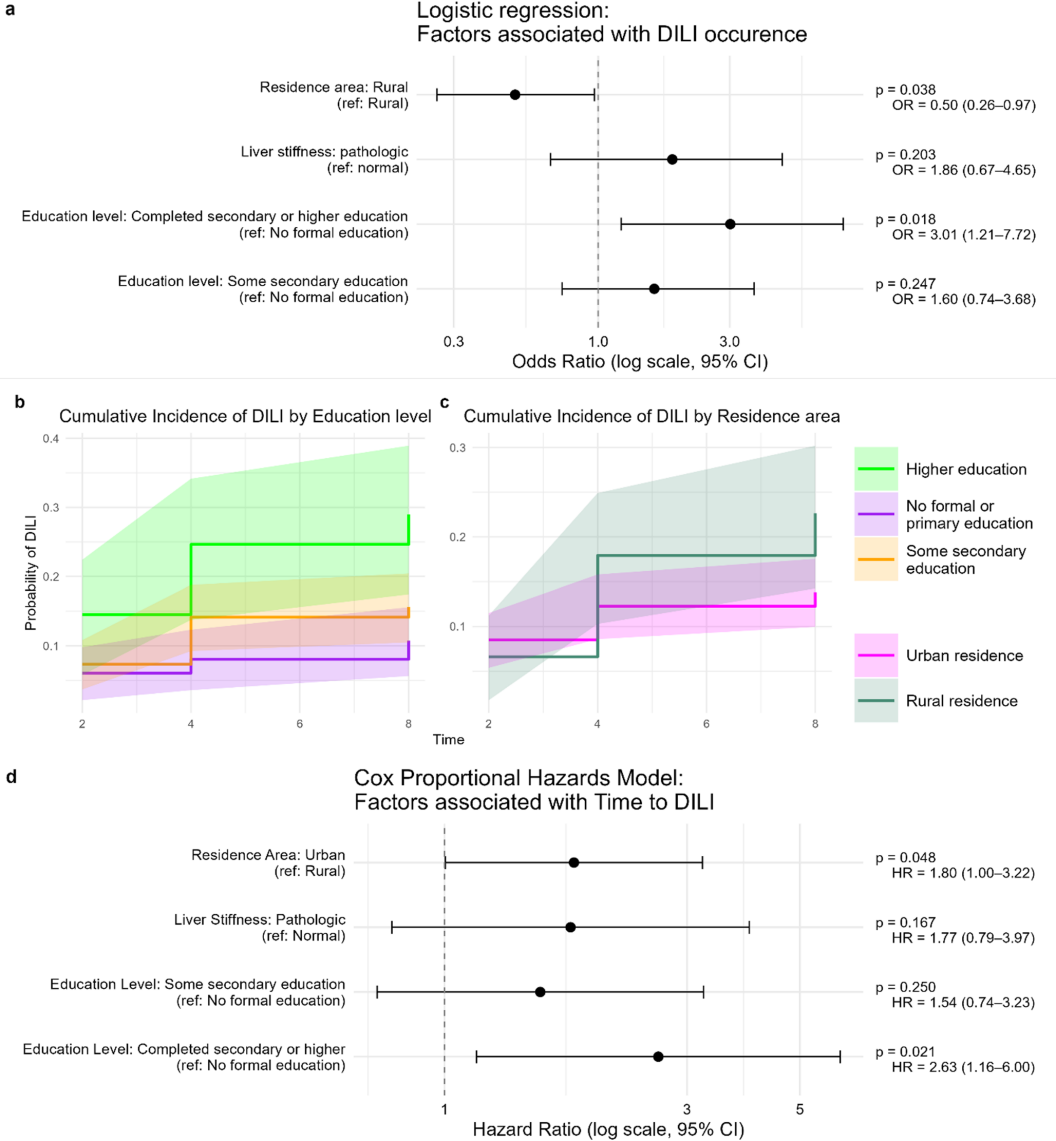
**a**: Logistic regression model assessing risk factors for the occurrence of drug induced liver injury in the cohort. **b** and **c**: Cumulative incidence of drug-induced liver injury occurrence in subgroups according to educational status and residence area of participants. **D**: Cox Proportional Hazard Model assessing the impact of predictor variables on the time to drug-induced liver injury occurrence. DILI: drug-induced liver injury; OR: Odds ratio; CI: confidence interval; HR: Hazard ratio;

Logistic regression analysis revealed that urban residence was significantly associated with an increased risk of DILI occurrence (Odds ratio [OR] 2.00, 95% Confidence interval [CI] 1.03–3.84, *p* = 0.038, Fig. 4a). Additionally, individuals who had completed secondary or higher education demonstrated a higher risk of DILI compared to those with no formal education (OR 3.01, 95% CI 1.21–7.72, *p* = 0.018). In contrast, preexisting pathologic liver stiffness did not show a statistically significant association with DILI occurrence (*p* = 0.203). Cumulative incidence plots indicated that the probability of developing DILI over time was highest among individuals with completed secondary or higher education, while those with no formal or only primary education had the lowest probability. Similarly, urban residents exhibited a greater cumulative incidence of DILI compared to their rural counterparts (Fig. 4b-c). Cox proportional hazards modeling further supported these findings, showing that urban residence (Hazard ratio [HR] 1.80, 95% CI 1.00–3.22, *p* = 0.048) and completed secondary or higher education (HR 2.63, 95% CI 1.16–6.00, *p* = 0.021) were significantly associated with a shorter time to DILI onset. No significant associations were observed for pathologic liver TE measurements in relation to time to DILI (Fig. 4d).

**Fig. 5:**
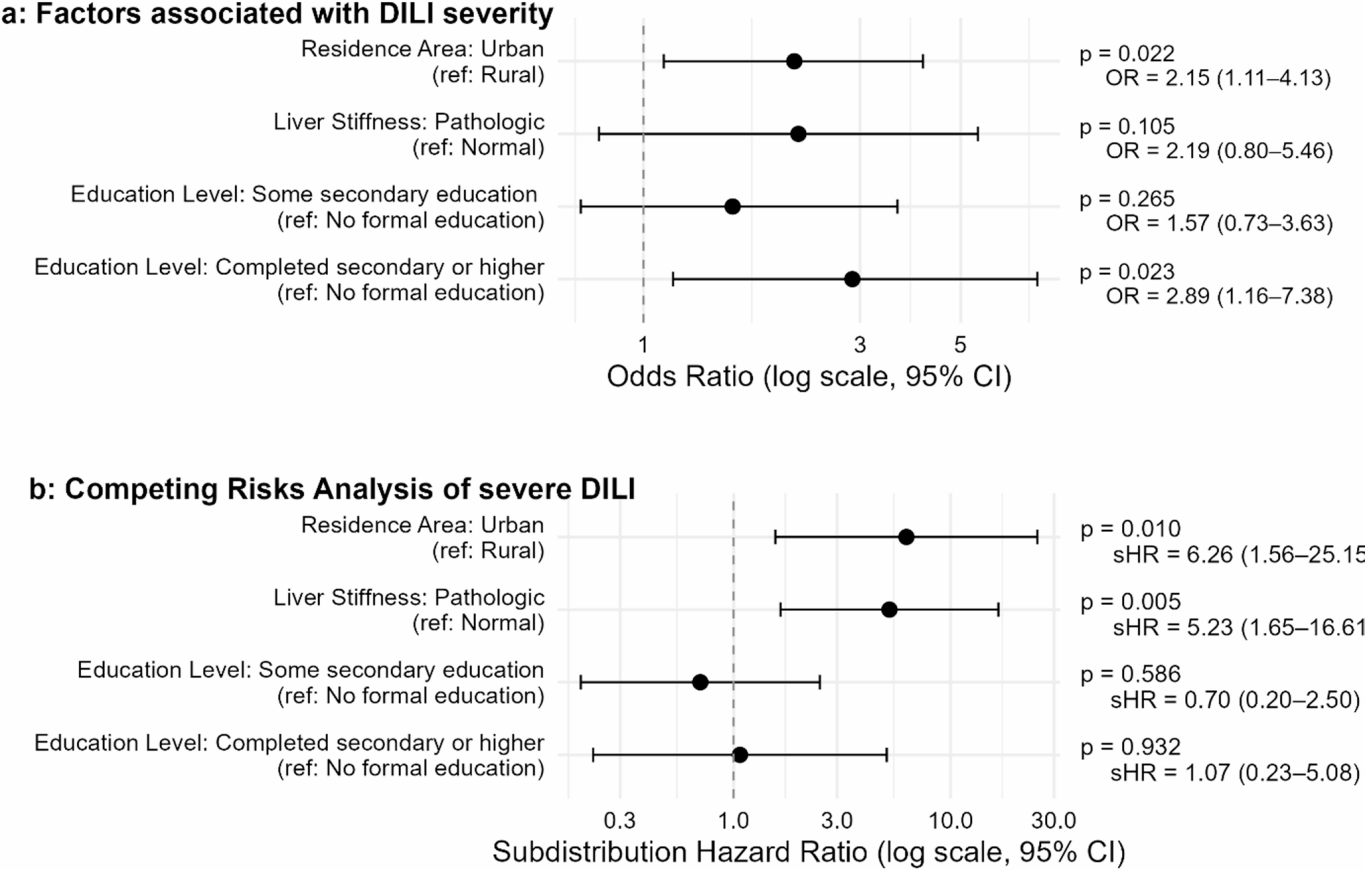
Factors associated with severity of drug-induced liver injury. **a**: Ordinal regression model assessing risk factors for non-severe and severe drug-induced liver injury as ordinal dependent variable. **b**: Fine and Gray time-to-event (severe drug-induced liver injury ) model accounting for competing risks (non-severe drug-induced liver injury). DILI: drug-induced liver injury; OR: Odds ratio;.sHR: subdistribution hazard ratio;

Ordinal regression analysis identified that both completed secondary or higher education (OR 2.89, 95% CI 1.16–7.38, *p* = 0.023) and urban residence (OR 2.15, 95% CI: 1.11–4.13, *p* = 0.022) were significantly associated with increased severity of DILI, whereas pathologic liver stiffness did not reach statistical significance (*p* = 0.105, Fig. 5a). Cumulative incidence plots demonstrated that individuals with higher education levels and those residing in urban areas experienced a higher probability of severe DILI events over time compared to their counterparts with lower education or rural residence (Suppl. Figure 1). Further, competing risks analysis using a Fine and Gray time-to-event subdistribution hazard model revealed that urban residence (subdistribution hazard ratio [sHR] 6.26, 95% CI 1.56–25.15, *p* = 0.010, Fig. 5b) and pathologic liver stiffness (sHR 5.23, 95% CI: 1.65–16.61, *p* = 0.005) were significantly associated with increased risk of a shorter time to severe DILI. Education level did not show a statistically significant effect in this model.

## Discussion

Drug-induced liver injury (DILI) is one of the most frequent and clinically relevant complications of tuberculosis treatment (TBT), with substantial implications for treatment adherence, therapeutic outcomes, and TB control efforts in high-burden settings. This prospective observational study provides comprehensive insight into the incidence, timing, and risk factors associated with DILI in Ethiopian TB patients undergoing standard TBT (Fig. 1). The observed cumulative incidence of DILI exceeded 16% within the first eight weeks, aligning with previously reported global and Ethiopian data and falling within the mid-range of DILI rates observed in TB patients [11–13, 19, 20, 27], thereby reflecting the occurrence of DILI in overlapping time windows among some patients and highlighting the dynamic nature of hepatic injury during early TBT. Notably, severe DILI affected nearly one in four patients (4.2%), with the majority of events occurring within the first two weeks of treatment initiation, emphasizing the need for early and intensive liver function monitoring (Fig. 2). We analyzed responses from a comprehensive questionnaire to identify variables potentially associated with the development of DILI. The cohort covered a wide age range, with a median age of 30 years and an almost equal sex distribution (43.16% female vs. 56.84% male; Table 2). Several sociodemographic and clinical factors were significantly associated with both the occurrence and severity of DILI. Urban residence emerged as a consistent and strong predictor across logistic, Cox, and Fine and Gray competing risks models (Figs. 4 and 5, Suppl. Figure 1). This may reflect differential environmental exposures, co-medication profiles, dietary patterns, or access to diagnostic and health services. Intriguingly, higher educational attainment was also associated with increased DILI risk and severity in multiple models. It is unlikely that a direct causal effect exists between higher education and DILI risk. While unexpected, this may reflect unmeasured lifestyle factors such as greater health-seeking behavior, self-medication practices, or reporting bias. These findings underscore the need to further explore social determinants of hepatotoxicity in TB treatment programs (Figs. 4 and 5, Suppl. Figure 1). Conversely, neither age, sex, smoking, nor self-reported alcohol use or chronic co-morbidities were associated with DILI in this cohort, which stands in contrast to previous studies [11–13, 19].

Interestingly, patients who declined to answer questions regarding their medical history - particularly about substance use (khat, alcohol, tobacco) or chronic illnesses - showed a significantly increased risk of DILI (Fig. 3, Suppl. Table 4). While the reasons remain unclear, it is conceivable that these individuals had unreported or unregulated consumption of hepatotoxic substances and were afraid of social stigmatization. Supporting this hypothesis, non-responders also had significantly higher liver stiffness measurements (LSM) on transient elastography (TE) at baseline, suggesting potential subclinical liver injury or advanced liver disease in this subgroup (Fig. 3). Notably, a majority of the non-responders were women, which may point to sociocultural factors influencing disclosure.

Importantly, TE-based liver stiffness measurements prior to TBT initiation revealed subclinical liver fibrosis or cirrhosis in a subset of patients, and the presence of elevated liver stiffness was significantly more common in those who developed severe DILI. Although liver stiffness was not associated with general DILI risk or time to onset in multivariable models, it was independently linked to an increased risk of early-onset severe DILI in the competing risks model (sHR 5.23, *p* = 0.005; Fig. 4). Notably, a lack of response to questions on alcohol or khat use was also significantly associated with higher liver stiffness values (LSM) on transient elastography at baseline, suggesting potential unreported liver injury or advanced liver disease in this subgroup. Unlike previous reports, in our cohort chronic HBV, HCV, or HIV infections, previously identified as a risk factor in an Ethiopian cohort, were not associated with an increased risk of DILI in our study [7, 19] (Suppl. Table 5). Patients with HBV and HIV were well represented in our study (HBV: 8.25%; HIV: 13.94%), while the prevalence of chronic HCV was underrepresented (< 1%). However, chronic viral infections - particularly HBV and HIV - were significantly associated with elevated liver stiffness values prior to treatment initiation, further supporting the presence of underlying liver fibrosis. This supports previous reports suggesting that underlying liver disease may predispose to more severe hepatic outcomes during hepatotoxic therapy and highlights the value of non-invasive liver assessment in TB patients prior to TBT [12]. The study also investigated genetic variants in the genes of the hepatobiliary transport proteins MDR1, MDR3, and BSEP, which were previously associated with cholestatic liver diseases [23–26]. However, no significant associations between genetic variants and DILI outcome were observed (Table 1). Even the MDR1 SNP *rs1045642* that was identified as a risk factor for DILI under TBT in an Ethiopian study was not a significant risk factor in our study [27]. This may reflect limited sample size, low variant frequency, or the multifactorial nature of DILI, where environmental and metabolic factors may outweigh genetic predisposition in this setting. For instance, the MDR 1 SNP *rs1045642 (c.3435T > C)* was only detected homozygous in 5.2% of our patients (Table 3). Additionally, as no whole exome sequencing was performed, genetic variants associated with DILI but limited to previously insufficiently investigated ethnic groups such as the sub-Saharan African population might be overlooked. Such unidentified variants could serve as better predictors of DILI in this context.

This study has several limitations. First, the sample size may have limited the ability to detect subtle or low-frequency associations. Additionally, TE measurements were available, valid, and included (i.e., not excluded due to intermediate values) for only 298 patients, which may have limited the statistical power to detect significant differences. Second, as liver function tests were performed at predefined intervals, it is possible that transient or rapidly resolving DILI episodes may have gone undetected. Third, a more recent DILI case and severity definition published by an international expert group in 2011 incorporates additional laboratory parameters and repeated ultrasound examinations at each follow-up time point, which was not implemented in this analysis because of existing limitations at the study site [31]. Furthermore, reliance on self-reported data for substance use and medical history introduces the potential for recall or reporting bias, particularly for sensitive issues such as alcohol or khat use.

Taken together, these findings have important implications for TB management in resource-limited settings. First, they suggest that early liver monitoring, especially within the first two weeks, is critical for detecting and managing DILI. In this cohort, this is true particularly in patients with significant risk profiles such as urban residence, higher education, or elevated liver stiffness. Second, routine pre-treatment liver stiffness assessment via TE may help identify patients at higher risk of severe liver injury, allowing for closer follow-up or treatment modification. Third, while genetic screening may not yet be feasible or informative in this population, social and clinical stratification could aid in risk-tailored care.

Future research should explore the mechanistic basis of sociodemographic disparities in DILI risk and assess whether early detection and intervention strategies can reduce DILI-related morbidity. Longitudinal follow-up beyond the intensive treatment phase could further clarify long-term hepatic outcomes in TB patients receiving TBT.

## Electronic Supplementary Material

Below is the link to the electronic supplementary material.

Supplementary Material 1

## Data Availability

All data supporting the findings of this study are available within the paper and its Supplementary Information.

## Abbreviations

A: Adenine
ABCB: ATP binding cassette subfamily B
ALP: Alkaline phosphatase
ALT: Alanin aminotransferase
AST: Asparate aminotransferase
BSEP: Bile salt export pump
C: Cytosine
CLD: Chronic liver disease
DILI: Drug induced liver injury
EMB: Ethambutol
G: Guanine
HBV: Chronic hepatitis B virus
HCV: Chronic hepatitis C virus
HIV: Human immunodeficiency virus
HLA-B: Human leukocyte antigen-B
HTPP: Hepatic transport protein polymorphisms
IQR: Interquartile range
INH: Isoniazid
kPA: Kilopascal
LFT: Liver function test
LMIC: Low- and middle-income countries
MDR: Multidrug resistance
PZA: Pyrazinamide
RR: Relative risk
RMP: Rifampicin
SNP: Single nucleotide polymorphism
T: Thymine
TB: Tuberculosis
TBT: TB treatment
TE: Transient elastography
ULN: Upper limit of the normal range
WHO: World Health Organization
