# Supplementary material for: Genetic and clinical risk factors for anti-tuberculosis drug-induced liver injury: insights from a prospective cohort study in central Ethiopia
Source: Infection. 2025 Sep 4;53(6):2833–46. doi: 10.1007/s15010-025-02632-7 (PMC12675616; doi:10.1007/s15010-025-02632-7)
Supplement: Supplementary file 1 — Supplementary Material 1 [file 15010_2025_2632_MOESM1_ESM.docx]

**Supplementary materials**

**Suppl. Table 1:** Standard treatment of Rifampicin-susceptible tuberculosis (adult dose) according to (5)

| **Drug** | **Daily dosage (maximum) in mg/kg body weight** |
| --- | --- |
| Isoniazid | 4-6 (300) |
| Rifampicin | 8-12 (600) |
| Pyrazinamide | 20-30 (not defined) |
| Ethambutol | 15-2ß (not defined) |

**Suppl. Table 2:** Primer used to investigate hepatobiliary transport protein polymorphisms; SNP: single nucleotide polymorphism; BSEP: bile salt export pump; MDR: multidrug resistance protein

| **SNP ID** | **Assay ID** | **Reference Sequence** |
| --- | --- | --- |
| BSEP rs3815676 | C__27505204_10 | NM_003742.2: c.151-359A>G |
| BSEP rs7577650 | C___2034209_10 | NG_007374.1: g.1624C>T |
| BSEP V444A rs2287622 | C___15878656_10 | NG_007374.2: c.1331T>C |
| MDR1 rs1045642 | C___7586657_20 | NM_000927.4:c.3435T>C |
| MDR3 rs2109505 | C___1843468_30 | NM_018849.2: c.711A>T |
| MDR3 rs2302386 | C___1843475_20 | NM_018849.2: c.286+130T>C |
| MDR3 rs4148826 | C___1843464_10 | NM_018849.2: c.1006-128A>G |

**Suppl. Table 3:** Liver function tests (LFTs) according to follow up timepoint and baseline classification of liver enzymes

|  |  | Baseline | 2 weeks | 4 weeks | 8 weeks |
| --- | --- | --- | --- | --- | --- |
| Patients with normal baseline liver enzymes (n=368) | Aspartate transaminase (U/L) | 24 (19/33) | 24 (18/32) | 24 (19/30) | 23 (18/30) |
|  | Alanine transaminase (U/L) | 20 (15/29) | 22 (16/30) | 21 (16/30) | 20 (15/27) |
|  | Total bilirubin (mg/dL) | 0.50 (0.40/0.60) | 0.50 (0.40/0.60) | 0.50 (0.40/0.70) | 0.50 (0.40/0.60) |
| Patients with elevated baseline liver enzymes (n=56) | Aspartate transaminase (U/L) | 79 (62/107) | 27 (21/42) | 26 (20/39) | 26 (20/36) |
|  | Alanine transaminase (U/L) | 78 (45/125) | 28 (19/46) | 24 (18/37) | 24 (15/36) |
|  | Total bilirubin (mg/dL) | 0.60 (0.40/0.70) | 0.60 (0.50/0.80) | 0.60 (0.50/0.96) | 0.60 (0.40/0.80) |

**Suppl. Table 4:** Self-reported smoking, alcohol consumption, and Khat use habit. DILI: drug-induced liver injury; FDR: False Discovery Rate;

| **Variable** | **all** | **No DILI** | **Non-severe DILI** | **Severe DILI** | **p-value** | **FDR corrected post hoc p values** |
| --- | --- | --- | --- | --- | --- | --- |
| Smoking status |  |  |  |  | 0.001 | 0 vs 1: 0.929; 0 vs 2: 0.000; 1 vs 2: 0.003 |
| Yes | 22 (5.19%) | 19 (5.34%) | 2 (4%) | 1 (5.56%) |  |  |
| No | 374 (88.21%) | 319 (89.61%) | 45 (90%) | 10 (55.56%) |  |  |
| No information | 28 (6.6%) | 18 (5.06%) | 3 (6%) | 7 (38.89%) |  |  |
| Alcohol consumption |  |  |  |  | 0.001 | 0 vs 1: 0.286; 0 vs 2: 0.000; 1 vs 2: 0.001 |
| None | 235 (55.42%) | 205 (57.58%) | 24 (48%) | 6 (33.33%) |  |  |
| Moderate/Light | 105 (24.76%) | 88 (24.72%) | 13 (26%) | 4 (22.22%) |  |  |
| Heavy | 63 (14.86%) | 50 (14.04%) | 12 (24%) | 1 (5.56%) |  |  |
| No information | 21 (4.95%) | 13 (3.65%) | 1 (2%) | 7 (38.89%) |  |  |
| Khat consumption |  |  |  |  | 0.001 | 0 vs 1: 0.619; 0 vs 2: 0.000; 1 vs 2: 0.000 |
| Never | 346 (81.6%) | 294 (82.58%) | 45 (90%) | 7 (38.89%) |  |  |
| Sometimes | 30 (7.08%) | 24 (6.74%) | 3 (6%) | 3 (16.67%) |  |  |
| Frequent | 29 (6.84%) | 26 (7.3%) | 2 (4%) | 1 (5.56%) |  |  |
| No information | 19 (4.48%) | 12 (3.37%) | 0 (0%) | 7 (38.89%) |  |  |

**Suppl. Table 5:** Self-reported medical history and test outcomes for Hepatitis B; Hepatitis C and Human Immunodeficiency Virus. DILI: drug-induced liver injury; FDR: False Discovery Rate; HBV: Hepatitis B Virus; HCV: Hepatitis C Virus; HIV: Human Immunodeficiency Virus;

| **Variable** | **all** | **No DILI** | **Non-severe DILI** | **Severe DILI** | **p** | **FDR corrected post hoc p values** |
| --- | --- | --- | --- | --- | --- | --- |
| HBV status |  |  |  |  | 0.434 | 0 vs 1: 0.608; 0 vs 2: 0.666; 1 vs 2: 0.608 |
| Negative | 389 (91.75%) | 325 (91.29%) | 48 (96%) | 16 (88.89%) |  |  |
| Positive | 35 (8.25%) | 31 (8.71%) | 2 (4%) | 2 (11.11%) |  |  |
| HCV status |  |  |  |  | 1.000 | 0 vs 1: 1.000; 0 vs 2: 1.000; 1 vs 2: 1.000 |
| Negative | 420 (99.06%) | 352 (98.88%) | 50 (100%) | 18 (100%) |  |  |
| Positive | 4 (0.94%) | 4 (1.12%) | 0 (0%) | 0 (0%) |  |  |
| HIV status |  |  |  |  | 0.544 | 0 vs 1: 1.000; 0 vs 2: 0.656; 1 vs 2: 0.656 |
| Negative | 365 (86.08%) | 307 (86.24%) | 44 (88%) | 14 (77.78%) |  |  |
| Positive | 59 (13.92%) | 49 (13.76%) | 6 (12%) | 4 (22.22%) |  |  |
| Known Liver Disease |  |  |  |  | 0.001 | 0 vs 1: 0.098; 0 vs 2: 0.000; 1 vs 2: 0.000 |
| Yes | 1 (0.24%) | 0 (0%) | 1 (2%) | 0 (0%) |  |  |
| No | 406 (95.75%) | 346 (97.19%) | 49 (98%) | 11 (61.11%) |  |  |
| No information | 17 (4.01%) | 10 (2.81%) | 0 (0%) | 7 (38.89%) |  |  |
| Known Chronic Disease |  |  |  |  | 0.001 | 0 vs 1: 0.387; 0 vs 2: 0.000; 1 vs 2: 0.000 |
| Yes | 8 (1.89%) | 8 (2.25%) | 0 (0%) | 0 (0%) |  |  |
| No | 397 (93.63%) | 336 (94.38%) | 50 (100%) | 11 (61.11%) |  |  |
| No information | 19 (4.48%) | 12 (3.37%) | 0 (0%) | 7 (38.89%) |  |  |
| Past Tuberculosis Treatment |  |  |  |  | 0.966 | 0 vs 1: 1.000; 0 vs 2: 1.000; 1 vs 2: 1.000 |
| Yes | 303 (71.46%) | 251 (70.51%) | 38 (76%) | 14 (77.78%) |  |  |
| No | 51 (12.03%) | 44 (12.36%) | 5 (10%) | 2 (11.11%) |  |  |
| No information | 70 (16.51%) | 61 (17.13%) | 7 (14%) | 2 (11.11%) |  |  |
| Intake of other Drugs |  |  |  |  | 0.001 | 0 vs 1: 0.176; 0 vs 2: 0.000; 1 vs 2: 0.000 |
| Yes | 11 (2.59%) | 10 (2.81%) | 0 (0%) | 1 (5.56%) |  |  |
| No | 23 (5.42%) | 17 (4.78%) | 0 (0%) | 6 (33.33%) |  |  |
| No information | 390 (91.98%) | 329 (92.42%) | 50 (100%) | 11 (61.11%) |  |  |


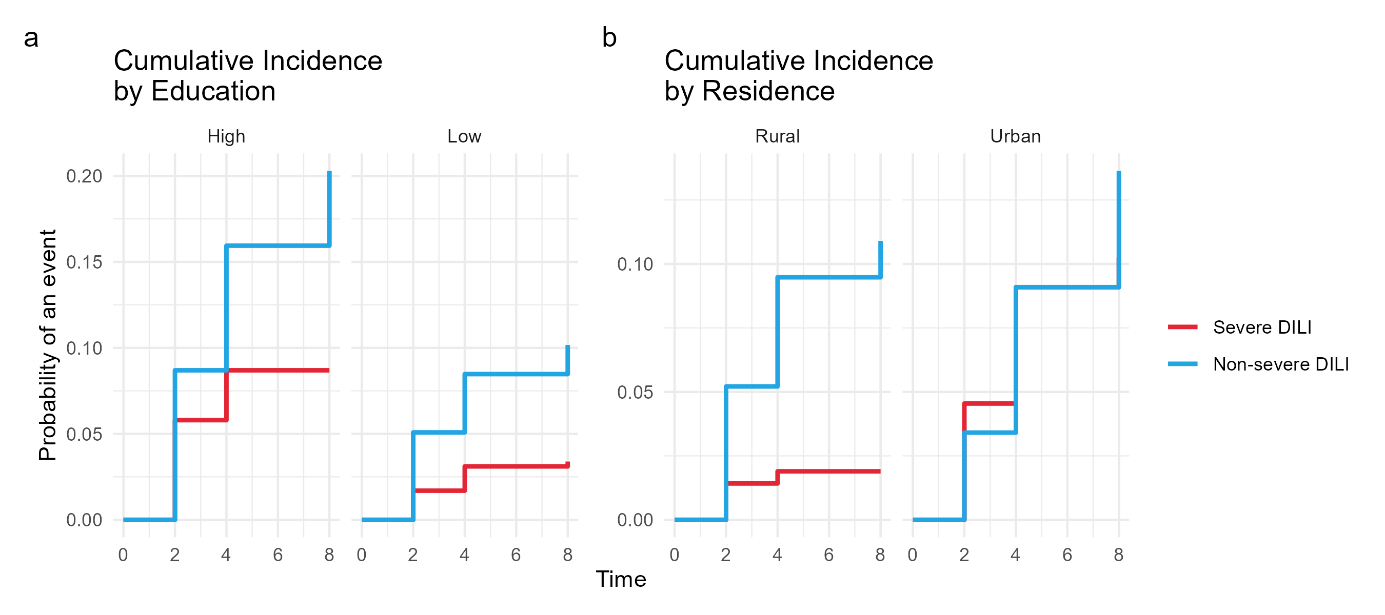


**Suppl. Figure 1:** a and b: Cumulative incidence of non-severe and severe drug-induced liver injury in subgroups according to educational status ("No formal or primary education", "Some secondary education" collapsed to “low” vs. "Completed secondary or higher education" as “High”) and residence area of participants. DILI: drug-induced liver injury;
